# How can gender be identified from heart rate data? Evaluation using ALLSTAR heart rate variability big data analysis

**DOI:** 10.1186/s13104-022-06270-2

**Published:** 2023-01-19

**Authors:** Itaru Kaneko, Junichiro Hayano, Emi Yuda

**Affiliations:** 1 Tohoku University Data-driven Science and Artificial Intelligence, Kawauchi 41 Aoba-Ku, Sendai, 980-8576 Japan; 2 Nagoya City University Graduate School of Medical Sciences, 1 Kawasumi Mizuho-Cho Mizuho-Ku, Nagoya, 467-8601 Japan

**Keywords:** Heart rate variability (HRV), Bio-signal processing, Biological big data analysis, Gender identification, Machine learning

## Abstract

**Objective:**

A small electrocardiograph and Holter electrocardiograph can record an electrocardiogram for 24 h or more. We examined whether gender could be verified from such an electrocardiogram and, if possible, how accurate it would be.

**Results:**

Ten dimensional statistics were extracted from the heart rate data of more than 420,000 people, and gender identification was performed by various major identification methods. Lasso, linear regression, SVM, random forest, logistic regression, k-means, Elastic Net were compared, for Age < 50 and Age ≥ 50. The best Accuracy was 0.681927 for Random Forest for Age < 50. There are no consistent difference between Age < 50 and Age ≥ 50. Although the discrimination results based on these statistics are statistically significant, it was confirmed that they are not accurate enough to determine the gender of an individual.

## Introduction

Jensen-Urstad et al. reported that heart rate variability has relation with gender and age [1]. Heart rate variability is known to be related to gender. If it is possible to accurately identify gender using fluctuations in heart rate as a clue, this can be major privacy concern of the volunteering subjects those provides heart rate variability data for the medical and scientific database. However, we believe that there have not yet been reliable results on how much gender can be determined from fluctuations in heart rate.

There are various studies on gender identification. Methods to discriminate male-female voices [2–5], facial and brain images [6, 7], discriminate from dynamic features such as gait and handwriting [8, 9], discriminate from text data such as names [10, 11], and discriminate from heartbeats sounds [11]. Those methods for gender identification based on physical characteristics have also been applied to transgender identification [12]. In this study, we confirmed how much gender can be identified using Holter ECG database.

## Main text

### Objective of the experiments

The collection and use of biological big data is becoming more and more important in recent years in the computerization of medical treatment. Heart rate variability big data is one of the most useful medical information among health medical information. ALLSTAR heart rate variability big data constructed as large-scale heart rate variability big data has collected more than 420,000 samples. We have already systematically performed various analyzes based on this data and have achieved many results [13–18]. On the other hand, for example, in the field of analysis of genetic information, it has been reported that information such as the area where an individual lives can be obtained from genetic information [19].

### Previous studies

Holter ECG has been evolving since the 2000s and is used today to measure many patients. For example, Jane et al. evaluated the automatic threshold-based detector [20]. Xuexiang et al. Performed CNN (Convolutional neural network) identification using Holter ECG data. They reported that VEB (ventricular ectopic beats) and SVEB (supraventricular ectopic beats) detection obtained a high detection rate of 97.5% or more [21]. Agelink, Yukishita et al. We investigated gender differences in heart rate variability and reported that there was a slight gender difference in heart rate variability [22, 23]. Gender determination from heart rate variability is not impossible, but generally it is not so accurate.

### Experimental method

ALLSTAR is 24-h Holter ECG big data. This big data contains more than 420,000 heart rate variability samples, each heart rate variability sample contains 24-h ECG record. The number of subjects was 429,308, including 861 subjects who measured ECG twice. In experiments with 71,264 samples for subjects under the age of 50, the number of subjects was 71,126, which included 138 subjects who measured ECG twice. No subject measured ECG more than two times.

Statistical features used in the analysis. HR is the 24-h mean value of the R-R interval of continuous sinus rhythm, SDNN is the standard deviation, and rMSSD is the rms (root mean square) of the difference of R-R intervals. The changes in the R-R interval are frequency-analyzed as a sample series, and the components are extracted for each ULF (ultra-low frequency, 0 to 0.0033 Hz), VLF (very low frequency, 0.0033 to 0.04 Hz), LF (low frequency, 0.04 to 0.15 Hz) and HF (high frequency, 0.15 to 0.4 Hz). Furthermore, DFA1 (Detrended fluctuation analysis 1) and DFA2 (Detrended fluctuation analysis 2) are calculated by detrended fluctuation analysis. In this time, we conducted a gender identification experiment based on these statistical indicators as 10-dimensional indicators.

Evaluation method used for comparison. In this time, we compared 4 types of classification identification methods. As classification method, we verified three types of classification methods: k-means and identification methods: random forest and SVM. Using all 428,302 data as a sample, test data was set to 60%, and it was obtained using the library of scikit-lab. Fourfold cross validation was performed to four different divisions, and the average was calculated. We had already confirmed this setting gives reliable result in our previous studies. K-fold cross validation is commonly used method to increase the statistical precision from given limited of dataset to be used for training and testing data. We had simply used widely used scikit-learn based API (Application Interface) to perform it. We had chosen 60% test data. This is to balance the ratio of training data and test data for our evaluation for our purpose. For number of folds, we had confirmed fourfold gives the best result for our purpose which means larger k won’t give any major statistical precision. As regression analysis, Elastic net, Lasso, linear regression, and SVR (Support Vector Regression) were performed.

## Results

The experimental results for classification are shown in Table 1, results using all age groups, < 50 and ≥ 50.There are no consistent difference between Age < 50 and Age ≥ 50.

**Table 1:** Results using data of subjects in all ages, under 50 and over 50

Method	Age	Acc	Prec	Recall	F1
*k*-means	Under 50	0.512753	0.512799	0.999424	0.677815
	Over 50	0.540683	0.540742	0.999333	0.701759
Logistic regression	Under 50	0.513727	0.512773	0.998169	0.677501
	Over 50	0.540853	0.540859	0.999917	0.702002
Random forest	Under 50	0.681927	0.675664	0.727825	0.700766
	Over 50	0.655528	0.657664	0.757349	0.703986
SVM	Under 50	0.511716	0.511716	1.000000	0.676999
	Over 50	0.540859	0.540859	1.000000	0.702022

Number of all subjects: 428,302, Average age 65.16, Number of subjects Age < 50: 73,349, Average age 33.03 (± 13.12), Number of subjects Age ≥ 50: 347,555, Average age 71.94 (± 10.14). Statistical analysis using Fisher's exact test for < 50 and ≥ 50 showed that the probability of an event not related to age was low, and the results were considered to be related to age (p < 0.0001 for each method)

For the evaluation of classification using regression algorithms, assume two classes are male and female, and calculated r squared score. The results are shown in Table 2 The accuracy of the classification and discrimination method was 0.540 for k-means, logistic regression and SVM for Age ≥ 50. It is 0.681 for Random forest for Age < 50.

**Table 2:** Results using regression algorithm

Method	R squared score
Elastic net	− 0.000022
Lasso	− 0.000022
Linear reg	0.0825
SVR	− 0.00515

**Fig. 1:**
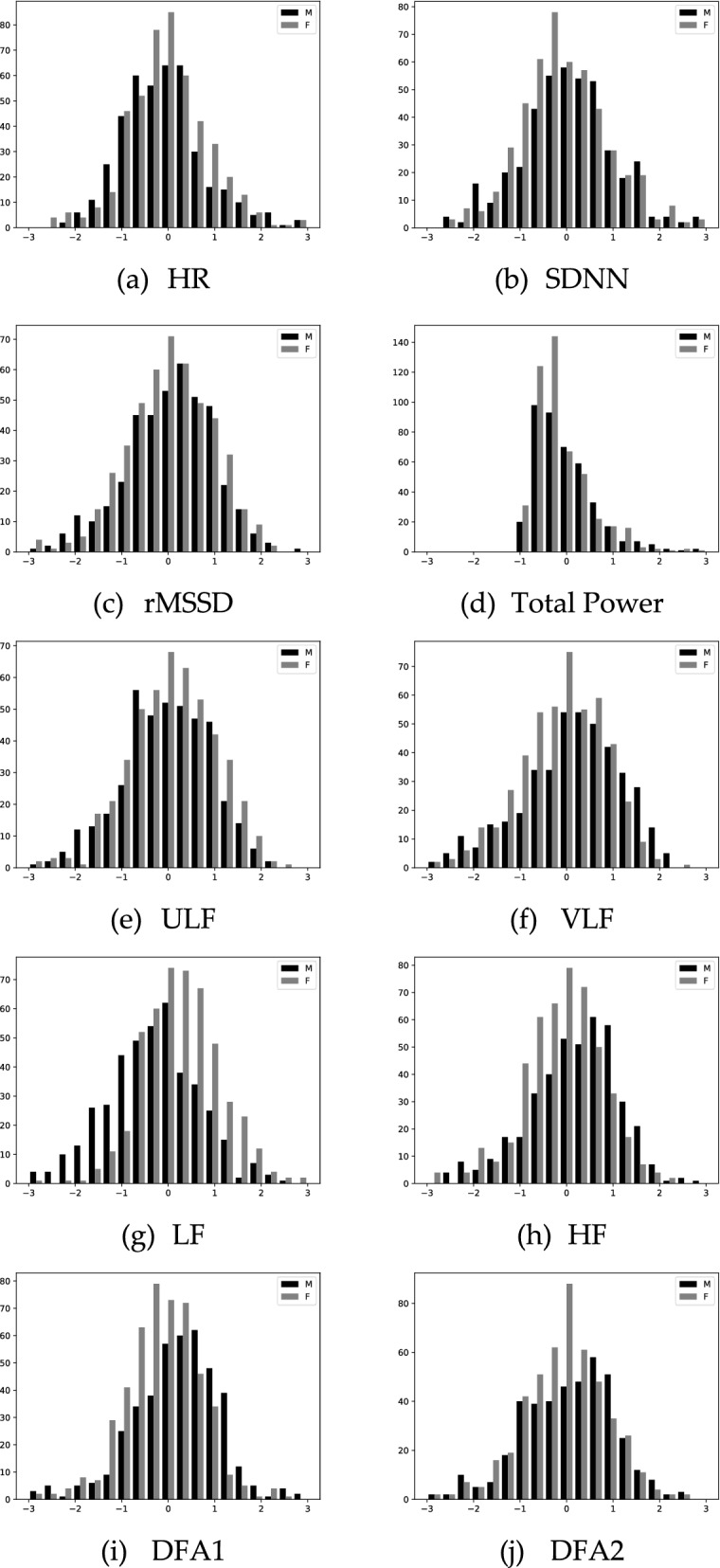
Comparison of histogram of the statistic index for male and female age under 50 Indicators are normalized within all subjects and then distribution of each group is calculated

The distribution of male and female parameters of the group under 50 years old is shown in Fig. 1. Differences in distribution are more pronounced under the age of 50.

## Discussion

In this study, we evaluated how precisely gender can be identified from heart rate variability data. Regarding the estimation of gender from heart rate variability, we were able to perform an estimation experiment using more data than in previous studies [24].

It was not so clear whether there is a gender difference in heart rate variability data. But it is a new finding that it was confirmed that there was a certain difference and revealed reliable performance index.

The presence or absence of age-related differences in classification ability may be due to sex hormone effects.

Ziegler et al. [25] discusses the normal range and reproducibility of statistical, geometric, frequency-domain, and nonlinear measurements of 24-h heart rate variability. Results show that, in healthy subjects, measurements of 24-h HRV are independent of sex and BMI, but strongly dependent on age and heart rate, and geometric parameters of HRV show high intra-individual reproducibility [25]. Voss et al. [26] found significant changes in indices according to gender in the frequency domain and correlation analysis, suggesting that the effects of gender and age should be considered when conducting HRV studies [26]. However, in our classification method, it was shown that it is difficult to classify gender from HRV.

Although previous studies have shown that gender labels are important for heart rate variability analysis, it is the first study to demonstrate the difficulty of accurately identifying gender in a short period of time using unlabeled data. As a future work, the effects of sex hormones on the autonomic nervous system, the effects of differences in behavioral characteristics between male/female on the autonomic nervous system, and the differences in health levels of subjects due to differences in medical examination behavior (medical examination thresholds) of gender would be beneficial if gender could be estimated.

## Limitations

Using larger number of data and seeing the results in different sample groups remains as further challenges. And deep learning is one of suitable method. However, the computational cost is large due to the huge amount of data, which is a limitation of this study.

## Data Availability

The data used in this analysis may be used for research purposes with the permission of the committee by submitting an application to the Allostatic State Mapping by Ambulatory ECG Repository (ALLSTAR https://allstar.jpn.org/), if necessary. The data used in this analysis can be used for research if permission is obtained from the committee.

## Abbreviations

HR: Average heart rate
SDNN: RR standard deviation
rMSSD: Differential RMS
ULF: Ultra Low Frequency (〜0.003 Hz)
VLF: Very Low Frequency (0.0033〜0.04 Hz)
LF: Low Frequency (0.004〜0.15 Hz)
HF: High Frequency (0.15〜0.4 Hz)
DFA1: Detrended Fluctuation Analysis 1
DFA2: Detrended Fluctuation Analysis 2
